# Mechanism of Qihuang needle therapy in the management of tic disorders: a clinical trial protocol

**DOI:** 10.3389/fneur.2023.1036453

**Published:** 2023-04-20

**Authors:** Yuyuan Tang, Jun'e Wu, Zhirui Xu, Baochao Fan, Xiangli Li, Bingxu Jin, Chunzhi Tang

**Affiliations:** ^1^Clinical Research and Big Data Laboratory, South China Research Center for Acupuncture and Moxibustion, Medical College of Acu-Moxi and Rehabilitation, Guangzhou University of Chinese Medicine, Guangzhou, China; ^2^Department of Reproductive Health, The Fourth Clinical Medical College of Guangzhou University of Chinese Medicine, Shenzhen, China; ^3^Department of Traditional Chinese Medicine, Guangzhou First People's Hospital, Guangzhou, China; ^4^Department of Rehabilitation, Panyu Hospital of Chinese Medicine, Guangzhou, China

**Keywords:** multi-omics analysis, clinical trial protocol, acupuncture, Qihuang needle therapy, tic disorders

## Abstract

**Background:**

Qihuang needle therapy is a newly developed acupuncture therapy to treat tic disorders in clinical practice. However, the mechanism to reduce tic severity remains unknown. Changes in intestinal flora and circulation metabolites are perhaps the potential pathogenesis of tic disorders. As a result, we present a protocol for a controlled clinical trial using multi-omics analysis to probe the mechanism of the Qihuang needle in managing tic disorders.

**Methods:**

This is a matched-pairs design, controlled, clinical trial for patients with tic disorders. Participants will be allocated to either an experimental group or a healthy control group. The main acupoints are Baihui (GV20), Yintang (EX-HN3), and Jueyinshu (BL14). The experimental group will receive Qihuang needle therapy for a month, while the control group will receive no interventions.

**Expected outcomes:**

The change in the severity of the tic disorder is set as the main outcome. Secondary outcomes include gastrointestinal severity index and recurrence rate, which will be calculated after a 12-week follow-up. Gut microbiota, measured by 16S rRNA gene sequencing; serum metabolomics, assessed *via* LC/MS; and serum zonulin, assessed by enzyme-linked immunosorbent assay (ELISA), will be used as biological specimen analysis outcomes. The present study will investigate the possible interactions between intestinal flora and serum metabolites and the improvement of clinical profiles, which may elucidate the mechanism of Qihuang needle therapy for tic disorders.

**Trial registration:**

This trial is registered at the Chinese Clinical Trial Registry (http://www.chictr.org.cn/). Registration number: ChiCTR2200057723, Date: 2022-04-14.

## 1. Introduction

Tic disorders are neurodevelopmental disorders characterized by recurrent motor and/or vocal tics (1). The Diagnostic and Statistical Manual of Mental Disorders, Fifth Edition (DSM-5) (2), defines the following five tic disorders: provisional tic disorder; persistent (chronic) motor or vocal tic disorder; Tourette's disorder (also known as Tourette's syndrome [TS]); other specified tic disorder; and unspecified tic disorder. Tic disorders are common in China, with a prevalence of transient tic disorder (TTD), chronic tic disorder (CTD), and Tourette syndrome (TS) of 1.7, 1.2, and 0.3%, respectively (3).

Tics usually begin between the ages of 3 and 8 years and become worse between the ages of 8 and 12 years for most patients. The first tic symptoms are usually simple motor tics, restricted to one muscle or a single muscle group. Tics then progress to involve more muscle groups or present with mimicking. Simple vocal tics, demonstrating meaningless sounds, usually occur after motor tics. For children or adolescents, the features of tics wax and wane in severity and frequency, and new tic symptoms replace, combine with, or even exacerbate the old ones (4, 5). Academic stress and family pressure may increase the incidence of tic disorders among school-age children. Excessive attention from parents, ridicule from peers, or incomprehension from teachers contribute to the aggravation of tics. Despite reports that tics tend to mitigate spontaneously throughout adolescence and reach complete remission in early adulthood, tics do not always remit within a year in most children with recent-onset tic disorders, and recurrence may occur even after long-term remission among patients with CTD (6). Attention-deficit hyperactivity disorder, obsessive-compulsive disorder, and autism spectrum disorder are the most common psychiatric comorbidities that impair social, behavioral, or emotional functioning (7).

Psychoeducation is recommended as the initial intervention, and behavior therapy is recommended as a first-line intervention for children and adults with CTD or TS (8). Nevertheless, education and behavior therapy are aimed at older patients or children with insufficient evidence to prove effectiveness and safety (9). Pharmacotherapy is recommended as the second option but may cause considerable adverse effects (10). Furthermore, for refractory tic disorders, surgical interventions using deep brain stimulation have been suggested as an alternative option (11), but the efficacy and safety thereof are non-conclusive. Although various neural regulation therapies, such as repetitive transcranial magnetic stimulation (rTMS), cranial electrotherapy stimulation, and electroencephalographic biofeedback, have been used in clinical practice, the effects of such treatments are still a matter of debate (5). Thus, a therapeutic strategy for tic disorders is required.

A growing body of evidence has proven that acupuncture is an effective treatment for tic disorders (12, 13). Acupuncture, especially electroacupuncture or scalp acupuncture alone, demonstrates a positive effect in terms of total clinical effectiveness rate, the incidence of adverse events, and recurrence. Currently, common methods of acupuncture treatment for tic disorders include traditional Chinese medicine acupuncture, electroacupuncture, scalp acupuncture, and auricular acupuncture (14). However, these methods have some disadvantages, such as long-time needle retention, challenges in electric current control, or the necessity for the child to take the initiative. In addition, most patients are school-aged children with academic pressure, requiring short-course therapies with flexible treatment times.

Qihuang (QH) needle, invented by Dr. Zhenhu Chen, is a new acupuncture instrument developed from the Jiuzhen (Nine Needles) created in ancient times (15). Qihuang needle therapy (QHN therapy) absorbs the essence of the meridian sinew theory and the “five thorns” that originated from the Inner Canon of Huangdi (16). QHN therapy has advantages compared with traditional acupuncture (17). First, it is less painful, with almost no pain when the needle punctures the skin. Second, the manipulation of the QH needle is swift, without needle retention. Third, point selection is simple and usually involves no more than five acupoints in one treatment session. Finally, the treatment period is shorter than traditional Chinese acupuncture, especially for intractable pain.

Recently, several cases that investigate the clinical efficacy of QHN therapy for various kinds of diseases such as low back pain (18, 19), acute gouty arthritis (20), and Parkinson's disease (21) have been reported. QHN therapy has been used in the field of pediatric neuropsychiatric disorders such as tic disorders (22) and spastic hemiplegic cerebral palsy (23). QHN therapy seems to be a potential treatment method for tic disorders, and further studies exploring its efficacy and mechanisms are needed.

Tic generation is caused by dysfunction of the cortico-basal ganglia neuronal networks (24). Changes in the gut microbiota could serve as a significant or moderating factor in the etiology of basal ganglia-associated diseases, such as tic disorders. A descriptive model of the microbiota-gut-brain axis in tic disorders has been reported (25, 26): genetic and environmental factors alter the gut microbial composition and its activity; disturbances in intestinal flora induce escalation of gastrointestinal permeability; and intestinal-derived molecules may pass into the peripheral circulation and permeate into the brain through the damaged blood-brain barrier, which may cause altered basal ganglia function. The gut microbial alteration may lead to other risk factors, including altered short-chain fatty acid levels, increased vagal stimulation, and the release of other bacterial metabolites. Zonulin is a human protein that could reversibly regulate intestinal permeability (27). Increased zonulin indicates impaired gut barrier integrity, and serum zonulin has been applied as a peripheral biomarker to assess human intestinal barrier permeability in neurological or mental diseases to explore the roles of dysbiosis and gut barrier integrity (28, 29). Under the hypothesis that intestinal-derived molecules penetrate the gut barrier into the peripheral circulation, corresponding molecular changes could be assessed *via* serum metabolomics. In addition, the interaction between the gut microbiome and serum metabolites can be explored to identify some previously unknown links between intestinal microbiota alterations, circulating metabolites, and physical disorders (30, 31).

Most studies regarding the mechanism of acupuncture therapy for tic disorders are conducted *via* animal model experiments, which focus on acupuncture regulating neurotransmitters (32, 33). Studies investigating the mechanism in the clinical context are insufficient. Recently, omics analyses have been used as prospective methods to probe acupuncture mechanisms in the clinical context. Using gut microbiome sequencing, acupuncture was found to modulate the structures and diversity of the gut microbiome, inhibit inflammation of the central nervous system (34, 35), gradually improve gut barrier function, and regulate neurotransmitters (36, 37) in various neuropsychiatric diseases. As for metabolomics assays, the potential mechanisms of acupuncture to treat neuropsychiatric disorders may be associated with modulating systematic inflammation and improving lipid metabolism (38–40). QHN therapy shows effectiveness in the treatment of tic disorders in clinical practice and is worth investigating to determine the specific mechanism. Nevertheless, omics studies of tic disorders are in their infancy (25, 41), with few studies on acupuncture. We aimed to determine how QHN therapy works in the treatment of tic disorders by investigating how it influences the gut microbiota and serum metabolites in children with tic disorders and to analyze possible correlations between clinical profiles and the changes in the gut flora and serum metabolites.

## 2. Methods and analysis

### 2.1. Study design and participants

This is a matched-pairs design, controlled, clinical trial with two groups (experimental group and healthy control group). Patients with tic disorders and age–gender-matched healthy children will be recruited through advertisements posted on web pages and display boards or through the outpatient system recommended by doctors at Panyu Hospital of Chinese Medicine (Guangzhou, China) and Foshan Fosun Chancheng Hospital (Foshan, China). Both hospitals are tertiary hospitals. All participants will be screened according to the inclusion and exclusion criteria. The guardians of eligible participants will sign a written informed consent (see Supplementary material 1) after a detailed explanation of the study by the investigators and will be provided with a baseline assessment. Participants are free to withdraw from the study at any time. This protocol was designed according to the Standard Protocol Items: Recommendations for Interventional Trials (SPIRIT) guidelines (42) and the Standard Protocol Items for Clinical Trials with Traditional Chinese Medicine 2018: Recommendations, Explanation, and Elaboration (SPIRIT-TCM Extension 2018) (43). The study was registered in the Chinese Clinical Trial Registry (ChiCTR2200057723).

### 2.2. Eligibility criteria

Participants must fulfill the diagnostic criteria for provisional tic disorder and persistent motor or vocal tic disorder according to the DSM-5 (2). Patients will be screened according to the inclusion and exclusion criteria listed in Table 1.

**Table 1:** Eligibility criteria.

	**TD patients**	**Healthy controls**
Inclusion Criteria	1. Aged between 4 and 18 years	1. Matched age, sex, family history of a psychiatric condition, parental marital status, mode of delivery and birth weight to the TD group^*^
	2. Meeting the diagnostic criteria of transient tic disorder and Persistent Motor or Vocal Tic Disorder according to the Diagnostic and Statistical Manual of Mental Disorders, Fifth Edition (DSM-5) (1, 2)	2. No current or history of head trauma, convulsions, epilepsy, growth and development disorders, mental illness, digestive system disease, abdominal operation or asphyxia at birth
	3. The course of a provisional tic disorder is more than 3 months	3. No current or history of medication of mental disorders especially drug influencing neurotransmitters
	4. The score of YGTSS >12	4. The mother is healthy during pregnancy (no history of infection, digestive system disease, trauma or taking any medication)
	5. Have not received acupuncture or moxibustion within 3 months, and drug withdrawal period ≥12 weeks	
	6. Guardians are willing to sign the informed consents	
Exclusion Criteria	1. Secondary tic disorders	
	2. Patients with severe diseases of important organ systems: liver and kidney damage, cardiovascular insufficiency, metabolic diseases, malignant tumors, etc., which may adversely affect our clinical observation	
	3. Obsessive-compulsive disorder, depression, Attention deficit hyperactivity disorder, anxiety, autism spectrum disorder, eating disorder, or a history of substance abuse	
	4. Epilepsy, Huntington's disease, rheumatic chorea, hepatolenticular degeneration, substantia nigra degeneration, psychogenic tics and other extrapyramidal disorders	
	5. The use of antibiotics, probiotics, high-dose vitamin or immunomodulatory medications within 4 weeks before the fecal sample collection	
	6. Conditions like any infective diseases or other severe disorders that may influence the gut microbiota	
	7. Ulcers or surgical scars on the acupoint area	
	8. Children who are participating or participated in other clinical trials within 1 month	
Withdrawal criteria and management	1. Misdiagnosis, violation of the inclusion criteria	
	2. Self-use of prohibited treatment	
	3. Poor cooperation with the established treatment or data collection procedures	
	4. Serious adverse events or life-threatening diseases or an acute progression of the disease occurs during the trial	
	5. Obvious discomforts arise because of the therapy	
	6. Participants request withdrawal voluntarily	

American Psychiatric Association (1).

* Healthy control matching procedure: To select a healthy control group comparable in size to the experimental group. The criteria used for this procedure (with match tolerance) are as follows: (1) age (±1 year), (2) sex (exact match), (3) family history of the psychiatric condition (exact match), (4) parental marital status (exact match), (5) mode of delivery (exact match), and (6) birth weight (±100 g). We chose to match these variables as they may be the risk factors influencing the development of tic disorders.

### 2.3. Sample size

Considering that the focus of omics studies is the mechanism of the intervention effect, the sample size estimation of our exploratory research is distinctive compared with classical randomized control trials. Given previous literature regarding the omics analysis of acupuncture (44–46), at least 15 participants in each group are required. Thus, we set at least 15 participants in each group in the present design. Allowing for a 20% dropout rate, we plan to recruit 40 participants (20 participants per group).

#### 2.3.1. Recruitment strategies

**Figure 1:**
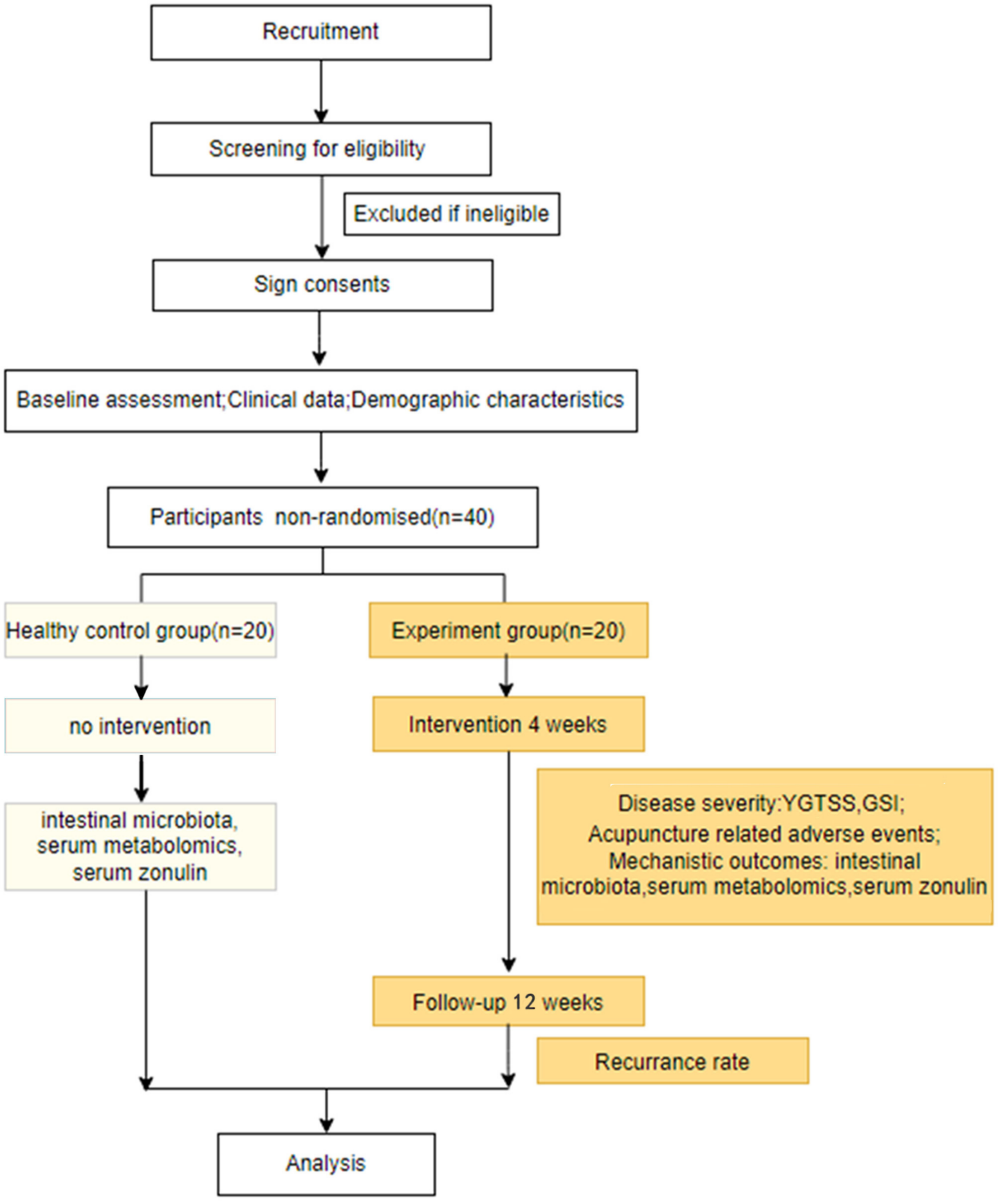
Flow diagram.

**Figure 2:**
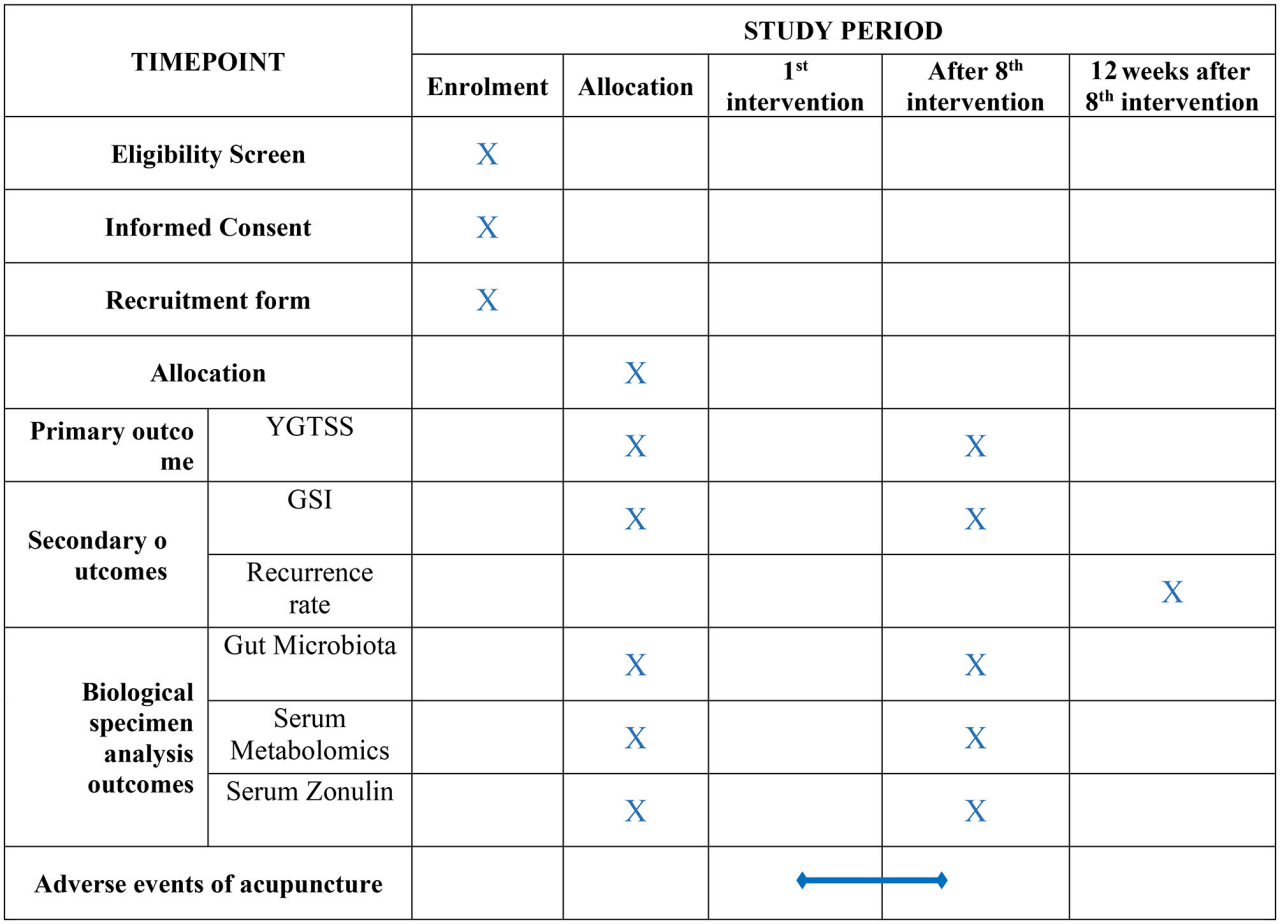
Schedule of trial enrollment, interventions, and assessments.

Participants were enrolled between April 2022 and March 2023. The current study utilized two primary resources to identify and recruit potential subjects: advertisements, including printed media such as roll-up banners and social media such as WeChat, and patients recommended by doctors. No registration fee was charged, and acupuncture treatments and scale assessments were provided free of charge. The flow of the trial is presented in Figure 1. The schedule of enrollment, interventions, and assessment, as well as visits for participants during the trial, is shown in Figure 2.

### 2.4. Intervention

To ensure the optimal effects of acupuncture stimulation, all treatments were performed by one certified acupuncturist with more than 3 years of experience in practice. Before the trial, special training regarding the process and standard operation of the treatment will be provided.

#### 2.4.1. Qihuang needle therapy

**Figure 3:**
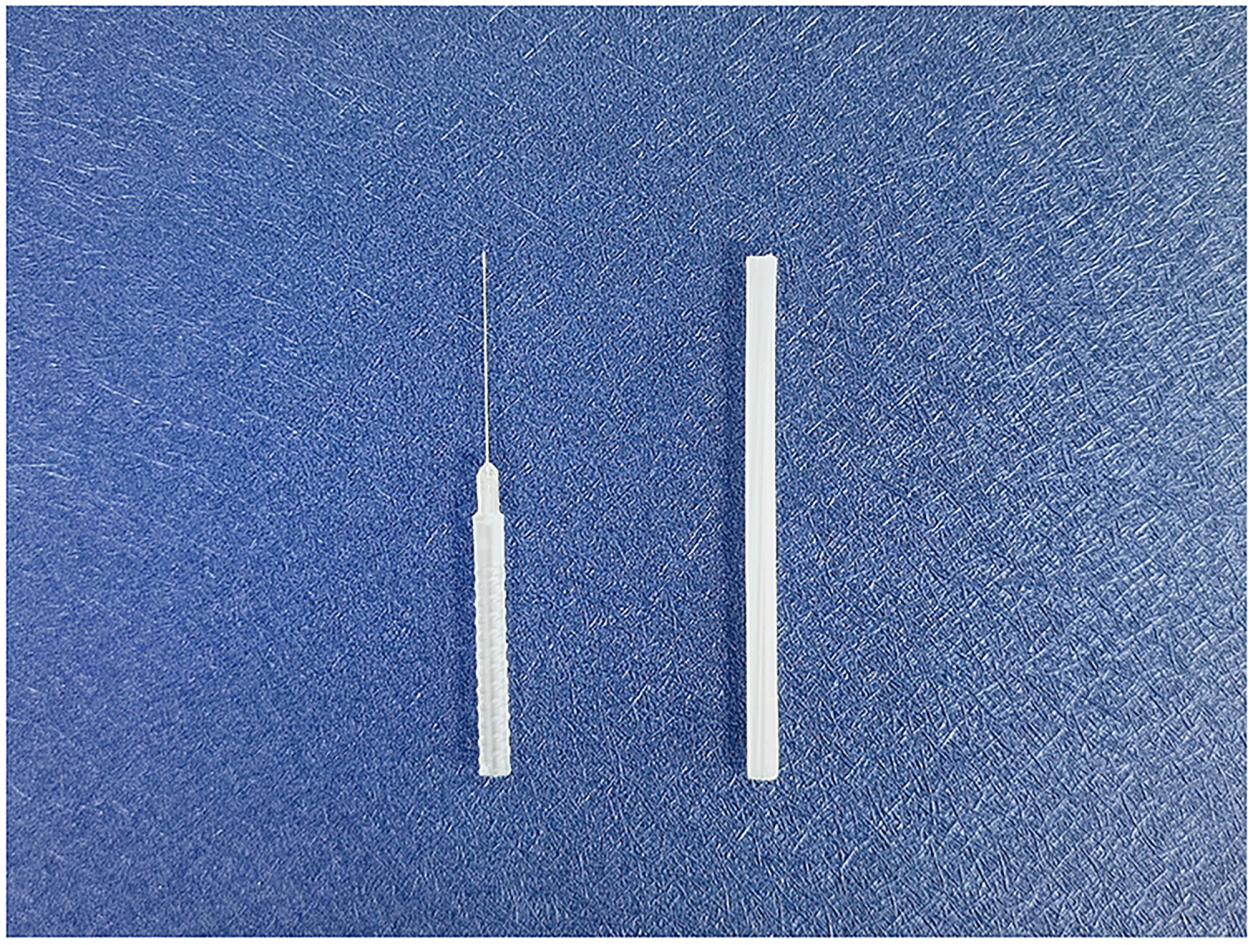
Qihuang needle **(left)** and the protective cap **(right)**.

The QH needle is divided into four parts as follows: the tip, the shaft, the handle, and the protective cap (Figure 3). Its tip is flat-bottomed, circular, and three-edged, ensuring safety and reducing stabbing pain at the moment of penetration (47). The needle body is a hollow tube with higher strength and toughness compared with common filiform needles. Its transparent and anti-slip handle facilitates the observation of hemorrhages, and the protective cap is made of disposable material that is non-toxic.

**Figure 4:**
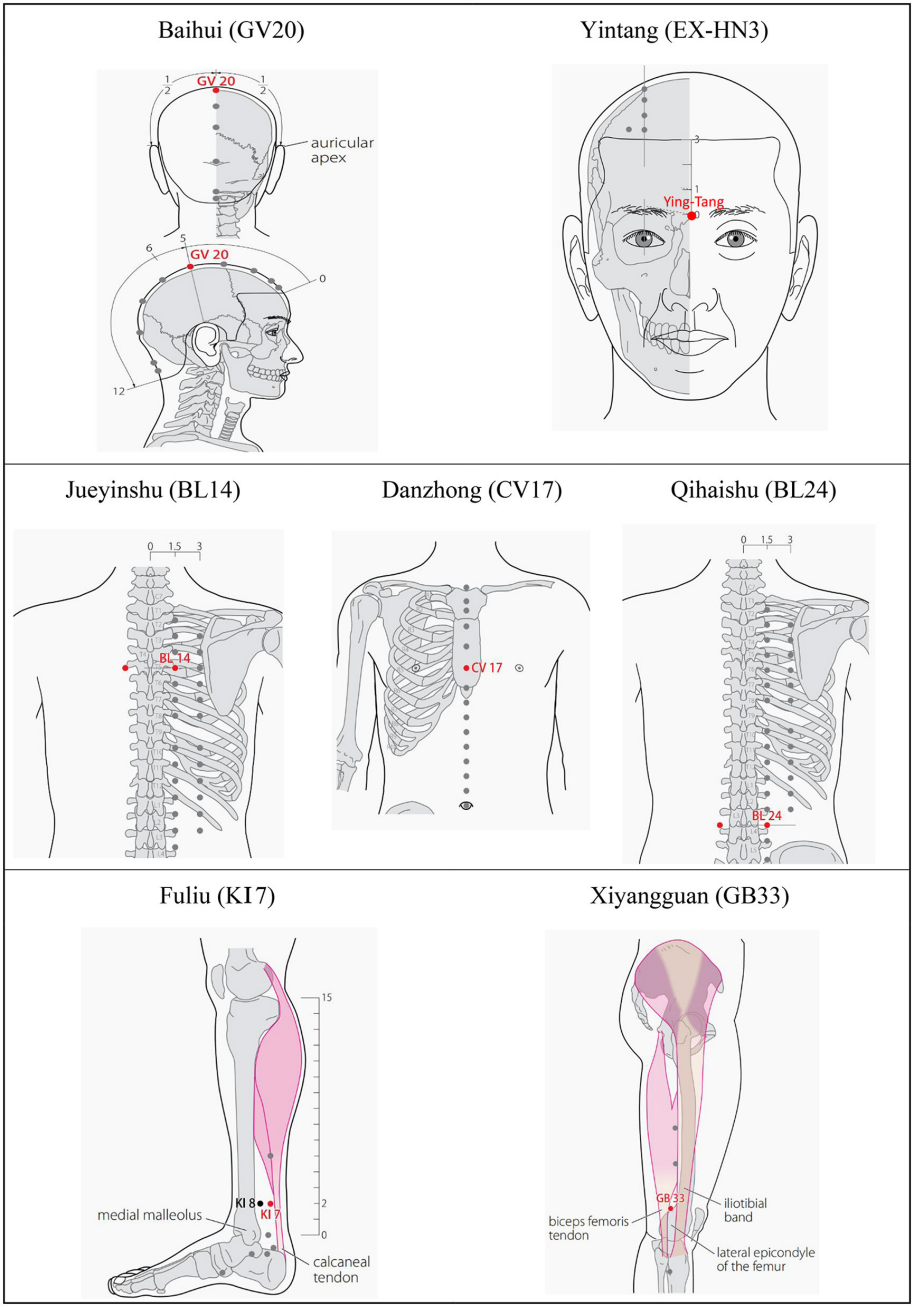
Location of acupoints.

Based on the meridian sinew theory and clinical experience, Baihui (GV20), Yintang (EX-HN3), and Jueyinshu (BL14) will be selected as the main acupoints. Xiyangguan (GB33), Fuliu (KI7), Danzhong (CV17), and Qihaishu (BL24) are additional acupoints. KI7 will be chosen for patients with leg twitches, and CV17 will be used where vocal tic disorders occur. BL24 is for waist movement disorder. BL14, GB33, KI7, and BL24 will be stimulated bilaterally. The acupuncture points are described in Table 2 and presented in Figure 4.

**Table 2:** Framework of the acupuncture point prescription.

**Acupuncture points**	**Description**
GV20: Baihui (p213)	On the head, 5B-cun superior to the anterior hairline, on the anterior median line
EX-HN3: Yintang	On the head, between the right medial end of the eyebrow and the left one
BL14: Jueyinshu (p116)	In the upper back region, at the same level as the inferior border of the spinous process of the fourth thoracic vertebra (T4), 1.5 B-cun lateral to the posterior median line
GB33: Xiyangguan (p188)	On the lateral aspect of the knee, in the depression between the biceps femoris tendon and the iliotibial band, posterior and proximal to the lateral epicondyle of the femur
KI 7: Fuliu (p139)	On the posteromedial aspect of the leg, anterior to the calcaneal tendon, 2 B-cun superior to the prominence of the medial malleolus
CV17: Danzhong (p228)	On the anterior medial line of the chest, at the midpoint between the two nipples, at the level of the fourth intercostal space
BL24: Qihaishu (p111)	In the lumbar region, at the same level as the inferior border of the spinous process of the third lumbar vertebra (L3), 1.5 B-cun lateral to the posterior median line.

The prescribed acupoints come from the WHO Standard Acupuncture Point Locations in the Western Pacific Region.

We aim to apply QH needles (patent no. ZL2015 20271867.0; 0.3 × 0.13 × 30 mm; QH; Chongqing Figure 3) in the acupuncture treatment. Manipulation (48) will be done as follows. After skin disinfection, the QH needles will be inserted vertically into the myofascial. Then, the needle will be directed toward the diseased site, and its handle will be gently moved from side to side at an angle of 15–30. When the “de qi” sensation (feelings of numbness, tingling, swelling, soreness, or muscle weakness) becomes obvious (~10 s after stimulation), the needle will be withdrawn. The pinholes will be pressed with a sterile, dry cotton ball without needle retention. As for the Baihui acupoint, the needle will be inserted perpendicularly into the galea aponeurotica, then withdrawn slightly, and the abovementioned manipulation will be performed. As for the Yintang acupoint, the needle will be inserted obliquely into the subcutaneous, and the abovementioned manipulation will be performed.

Participants in the experimental group will receive eight sessions of acupuncture treatment (two times a week for 4 weeks), and participants in the healthy control group will not receive the intervention.

### 2.5. Outcome evaluations

The clinical evaluations include the assessment of the severity of tics and gastrointestinal symptoms. The recurrence rate will be measured after a 12-week follow-up. The primary outcome measure will be the Yale Global Tic Severity Scale (YGTSS), and secondary outcomes will consist of the gastrointestinal severity index (GSI) and the recurrence rate. Biological specimen analysis outcomes will include changes in the composition of intestinal microbiota, serum metabolites, and serum zonulin. YGTSS and GSI will be assessed at baseline and after 4 weeks when the interventions have been completed. Data relating to the recurrence rate will be collected after a 12-week follow-up. Feces and serum samples will be collected at baseline and 4 weeks after the interventions. If sample collection is concurrent with a treatment, sample collection will be performed after all interventions have been completed. The feces sample will be collected as soon as possible after completing all interventions.

#### 2.5.1. Primary outcomes

##### 2.5.1.1. Change in YGTSS score

The YGTSS is the most widely used rating scale for tic assessment, both in clinical practice and research. It has the best evidence in the assessment of tic severity and indicates acceptable psychometric quality (49, 50). An experienced pediatric clinician carries out the YGTSS by asking the parents or guardians of the participants to answer the questions and by observing participant performance during visits to the clinic. The total score (maximum rating of 100) is the sum of the scores of motor and vocal tics and functional impairment. A YGTSS score < 25 indicates mild severity; 25–50, moderate; >50, severe.

#### 2.5.2. Secondary outcomes

##### 2.5.2.1. Change in GSI

The GSI, applied in the present study to rate the gastrointestinal signs and symptoms, was originally used in a 2006 study by Schneider et al. (51). This 9-item GSI rating scale was used to assess gastrointestinal symptoms in children with tic disorders, and its correlation with the gut microbiome was investigated (25). The rating scale consists of nine gastrointestinal signs and symptoms (constipation, diarrhea, average stool consistency, stool smell, flatulence, abdominal pain, unexplained daytime irritability, nighttime awakening, and abdominal tenderness), and the total possible severity score is 17, with a higher score corresponding to greater severity.

##### 2.5.2.2. Recurrence rate

The rate of recurrence will be calculated as the number of recurrences divided by the number of participant return visits.

#### 2.5.3. Biological specimen analysis outcomes

##### 2.5.3.1. Gut microbiota composition

Each participant will be asked to collect fresh feces in the morning, load them into a sterile container, and quickly place them into an ice box. The samples will be transferred to the laboratory with an ice pack for storage at −80°C. A DNA extraction kit will be used to extract the genomic DNA from the stool samples. The DNA will be purified, and its concentration will be detected using agarose gel electrophoresis. The DNA will be diluted with sterile water to 1 ng/nl for 16S rRNA amplification and sequencing. The 16S V3–V4 region (343F and 798R) or V4–V5 region will be amplified. DNA will be sequenced on the Illumina Novaseq 6000 platform, and paired-end reads (2 × 480 bp) will be generated. Sequencing and bioinformatics analysis will be conducted using OE Biotech (Shanghai, China) on the QIIME2 platform. Sequencing data analysis will be performed on amplicon sequence variants (ASVs).

##### 2.5.3.2. Serum metabolomics

The blood sample will be collected using a butterfly needle and syringe. In total, 3 ml of blood will be drawn into a serum tube and centrifuged, and 1 ml of aliquots will be transferred into three tubes and stored in a freezer at −80°C for the analysis of metabolomics. Metabolic profiling in both electrospray ionization (ESI) positive and ESI negative ion modes will be analyzed using the AB ExionLC System (ABSCIEX, Framingham, MA) coupled with a Q-Exactive quadrupole-Orbitrap mass spectrometer with a heat ESI source (Thermo Fisher Scientific, Waltham, MA). An ACQUITY UPLC HSS T3 column (100 × 2.1 mm, 1.8 μm) will be used in the positive and negative modes. The binary gradient elution system consists of (A) water (containing 0.1% formic acid, v/v) and (B) acetonitrile (containing 0.1% formic acid, v/v). The flow rate is 0.35 ml/min, and the column temperature is 45°C. A non-targeted metabolite analysis will be performed by Luming Biological Technology Co. Ltd. (Shanghai, China).

##### 2.5.3.3. Serum zonulin

Extraction and sample preparation will be performed according to the protocol described above. The concentrations of zonulin in all samples will be measured with a commercial kit (Elabscience, Houston, TX; Diaclone, Besancon, France; Boster Biological Technology, Pleasanton, CA; R&D Systems, Minneapolis, MN) using the enzyme-linked immunosorbent assay (ELISA) method.

##### 2.5.3.4. Adverse events

Acupuncture-related adverse events, including dizziness after acupuncture, local hematoma, infection, abscesses, and other unforeseen adverse events, will be recorded during all trial periods.

## 3. Data management and monitoring

Clinical data will be recorded in case report forms (CRFs) and imported into an electronic database. The CRFs and electronic databases will be locked after the study is completed. The personal data of all participants are kept anonymous and confidential. The original CRFs and other documents will be preserved securely at the South China Research Center for Acupuncture and Moxibustion at the Guangzhou University of Chinese Medicine. The relevant research team will supervise data quality, safety, and progress of the research.

### 3.1. Statistical analysis

#### 3.1.1. Clinical data and serum zonulin

For the clinical data, statistical analyses will be performed using SPSS Statistics 24.0 (IBM SPSS Statistics, Armonk, NY). Children in the experimental group will be matched to their peers in the healthy control group on age, sex, family history of a psychiatric condition, parental marital status, mode of delivery, and birth weight. Categorical variables will be described in the form of frequencies or percentages. Continuous variables will be presented as mean ± standard deviation or median and range. The chi-square test or Fisher's exact test will be used to compare the change in the categorical variables. Changes in YGTSS, GSI, and concentration of serum zonulin in the experimental group before and after treatment will be analyzed using paired sample *t*-test if the data conform to a normal distribution. The Wilcoxon paired-samples signed-rank test will be performed if the data do not conform to normal distribution or if there are differences in variance uniformity. Differences in concentration of serum zonulin between two matched groups at baseline will be analyzed with the above statistical methods. The results are based on two-sided tests, and a *P*-value of <0.05 will be considered statistically significant.

### 3.2. Gut microbiota

A *t*-test and Wilcoxon analysis will be used to identify the different microorganisms. A principal component analysis (PCA), a principal coordinate analysis, a non-metric multidimensional scaling, and a unweighted pair-group method with arithmetic means will be used to analyze beta diversity. An alpha diversity analysis will be performed using violin plots consisting of the chao1 index, the Shannon index, and the Simpson index. The COG family information and functional prediction of intestinal microbiota will be performed on Phylogenetic Investigation of Communities by Reconstruction of Unobserved States (PICRUSt2).

### 3.3. Serum metabolomics

#### 3.3.1. Metabolomic data preprocessing

Raw data are acquired using UNIFI 1.8.1, and the resulting mass spectra will be exported into Progenesis QI version 2.3 (Non-linear Dynamics, Newcastle, UK) for further analysis, including baseline filtering, peak identification, integral, retention time correction, peak alignment, and normalization, with the main parameters as follows: 5 ppm precursor tolerance, 10 ppm product tolerance, and 5% production threshold. Qualitative analysis will be performed *via* the Human Metabolome Database (HMDB), Lipidmaps (version 2.3), Metlin, the Electron Microscopy Data Bank, Protein Model Data Base, and self-built databases.

The extracted data will be removed if there are any peaks with a missing value of more than 50% or if the compounds with the resulting scores are below 36 (out of 60) points. A data matrix will be formed and imported into R. PCA will be carried out to observe the overall distribution among the samples and the stability of the whole analysis process.

#### 3.3.2. Differential expression analysis of metabolomics data

Orthogonal partial least squares discriminant analysis (OPLS-DA) and partial least squares discriminant analysis (PLS-DA) will be applied to seek differential metabolites between the healthy control and experimental group. Variable importance in projection (VIP) in the OPLS-DA model will be applied to investigate differentially expressed metabolites with biological significance. We will further perform a two-sided Student's *t*-test to verify whether the metabolite differences between the two groups are significant. If a metabolite has VIP values >1.0 and a *P*-value of <0.05, it will be selected as a differential metabolite. Functional pathway and network analysis *via* KEGG (https://www.kegg.jp/) database will be used to explore potential therapeutic targets of QHN therapy in children with tic disorders.

### 3.4. Correlation analysis of the microbiome and metabolome

Spearman's rank correlation coefficient will be calculated for all ASV-metabolite pairs and clinical parameters using the data across the same samples. A network analysis is to be performed on the interaction matrix using the fast greedy community algorithm in the igraph R package. This algorithm identifies subgraphs using direct optimization of a modularity score. Each discovered community is annotated according to the KEGG pathways of the metabolites within the community.

### 3.5. Trial status

Participants are currently being recruited for the present study.

## 4. Machine learning model on acupuncture response prediction

We will develop a machine-learning model to predict the acupuncture (QH needle) response. Transfer learning will be adopted. 16S rRNA, serum metabolomics, and clinical profile data will be downloaded from published data or public databases as source domains to generate a deep neural network (DNN) model. Data from the present study are set as the target domain to fine-tune the DNN model. An additional file shows the method in more detail (see Supplementary material 2).

## 5. Discussion

This is a matched-pairs design, controlled, clinical trial to investigate how QHN therapy relieves tic disorders *via* the modulation of gut microbiota and serum metabolites. To the best of our knowledge, the management of tic disorders is largely through psycho-behavioral therapy, which is not easy to access in many developing countries. Medicine is usually not the optimal selection because most patients are children. A search for a feasible and economic therapy for tic disorders should be promoted.

Acupuncture, as traditional Chinese medicine, has been widely accepted for its ready availability and lower cost. Its clinical efficacy in the treatment of pediatric neurological diseases, including cerebral palsy, autism spectrum disorders, and intellectual delay, has been proven. In addition, a growing body of research has proven that acupuncture shows good results in relieving tic symptoms, reducing recurrence rates, and causing fewer adverse events.

The main acupoints selected in the present protocol are under the sinew meridian theory based on traditional acupuncture. Tic disorders are categorized as meridian sinew disorders, and their pathogenesis is that qi and blood fail to nourish meridian sinew, resulting in trembling of the tendons and muscles, tics, and contractures. GV20 and EX-HN3 are the acupoints of the governor vessel that is located on the head or face. The governor vessel communicates with and governs all the yang meridians and runs into the brain. The three-foot yang and three-hand yang sinew channels start from four extremities and end at the face or the angle of the forehead. Acupuncturing at GV20 and EX-HN3 adjusts the yangqi of the entire human body and warms and nourishes the sinews. Furthermore, GV20 and EX-HN3 are widely used in neurological diseases, and it is reported that these two acupoints have positive effects in regulating neurotransmitter levels (52). BL14 is adjacent to the course of the meridian sinews of foot taiyang. Acupuncturing at BL 14 restores the balance of yangqi in meridian-sinews and is a transport point for the pericardium. It has been reported that BL14 is used in the treatment of tic disorders (53).

Qihuang needle therapy has advantages compared with traditional acupuncture among the pediatric population. First, it is less painful, with almost no pain when it penetrates the skin, which alleviates the fear of acupuncture in children. The manipulation of the QH needle is also swift, without needle retention, and point selection is usually simplified to no more than five acupoints in one treatment session. The design of the QH needle contributes to greater stimulation intensities at acupoints and meridians, which reduces treatment frequency. Because most patients are school-aged children, they do not have enough time to receive long-term acupuncture treatment as they are under academic pressure, and children do not usually tolerate long-term needle retention. These advantages help increase treatment adherence, yielding good efficacy.

16S rRNA has been used to identify the structure and diversity of gut microbes. Metabolomics has been applied to identify the metabolite profile of biofluid and the changes in small-molecule metabolites and related metabolite pathways in the disease state. The integration of both omics analyses is a prospective way to reveal the mechanism of acupuncture in the clinical context. Using 16S rRNA, some investigators have found that massage combined with acupuncture alleviated the severity of tics and improved gut permeability as well as intestinal flora (54). Aspartate/asparagine metabolism pathways and some metabolites were reportedly related to TS *via* metabolomics (41). It is feasible to explore the possible interactions between the metabolic phenotype and the alterations in the gut microbiota in tic disorders through the integrated application of 16S rRNA and serum metabolomics. Furthermore, potential therapeutic targets will be elucidated *via* multi-omics analysis.

16S rRNA sequencing from feces samples, UPLC-MS metabolomics from serum, and the assessment of serum zonulin will be performed in the present study to probe the potential mechanism and therapeutic targets of QHN therapy for tic disorders. The integration of 16S rRNA, metabolomics, and tic syndrome is likely to reveal the possible correlations between tic-associated metabolomes and tic-linked gut microbiomes, which may further explore the potential molecular mechanism underlying tic disorders. Furthermore, to investigate how QH needle treatment alters gut barrier function, serum zonulin will be used as an intestinal permeability assay in the present study.

In addition, to improve the clinical practice of acupuncture, we will further construct a prediction model based on multi-omics data and clinical profiles. Considering the small sample size, we will attempt to use the transfer learning method to develop a prediction model for personalized acupuncture treatment. Transfer learning is used to transfer knowledge from a generalized model to a more domain-specific model. It would enable the utilization of biological data published in the literature and reduce the demand for data collection when investigating new processes. Rogers et al. (55) showed that transfer learning is a highly data-efficient technique to predict the modeling of bioprocesses, a typically low-dimensional, small-data problem, but its usage in the biomedical field is scarce. In the present study, we will extract generalizable knowledge from well-understood published biological data, capturing essential process mechanisms to set a source model. We will then apply this understanding to characterize and screen novel samples and refine the model with our data. Our study is a new attempt to develop a prediction model for personalized acupuncture treatment.

Because most participants enrolled are school-aged children, they may have class during normal working hours, which may affect timely treatment. Therefore, we will make an appointment with parents after enrollment and keep in touch with them every week during the treatment sessions. In addition, we will do a 6-month follow-up to reduce the number of patients lost to follow-up. We will also maintain contact with the parents *via* WeChat or telephone and follow up through these channels.

This study has some limitations. There have been no acknowledged biomarkers for the assessment of tic severity. The rating of YGTSS is based on clinical experience in working with patients with tic disorder and their families, which is probably subjective. The sample size is small, and the follow-up duration is not very long.

## Ethics statement

The studies involving human participants were reviewed and approved by Medical Ethical Committee of Panyu Hospital of Chinese Medicine (Guangdong, China. Ethic Approval Number: 2022028) Foshan Fosun Chancheng Hospital (Guangdong, China. Ethic Approval Number: CYIRB-LCYJ-2022006-PJ-20221227). Written informed consent to participate in this study was provided by the participants' legal guardian/next of kin.

## Author contributions

YT, BJ, CT, and XL conceived and designed the study. YT and ZX drafted the manuscript and all authors revised the manuscript critically. YT, BF, and JW developed the statistical analysis of the trial and contributed to the content of the article. CT obtained funding. CT and XL are the study guarantors. All authors have read and approved the final manuscript.

## Supplementary material

Supplementary Material 1 Participant information letter.

Supplementary Material 2 Machine learning method.
