# Supplementary material for: Mechanism of Qihuang needle therapy in the management of tic disorders: a clinical trial protocol
Source: Front Neurol. 2023 Apr 20;14:1036453. doi: 10.3389/fneur.2023.1036453 (PMC10157291; doi:10.3389/fneur.2023.1036453)
Supplement: Supplementary Material 1 — Participant information letter. [file Data_Sheet_1.pdf]

Participant Information Letter

Project title: Study on the mechanism of Qihuang Needle Therapy in children with tic disorders based on multi-omics
Approval Number: 2022028/ CYIRB-LCYJ-2022006-PJ-20221227
Trial registration No. ChiCTR2200057723
Principal Investigator: Dr Chunzhi Tang

An invitation to participate in research

You are invited to participate in a project titled **"Study on the mechanism of Qihuang Needle Therapy in children with tic disorders based on multi-omics"**. You are being asked to take part in this project because you are aged between 4 and 18 years, healthy or diagnosed as tic disorder (transient/persistent motor/vocal tic disorder).

Please read this information carefully. Ask questions about anything that you do not understand or want to know more about. Before deciding whether to take part, you might want to talk about it with a relative or friend.

If you decide to take part in the research project, you will be asked to sign a consent form. By signing it, you are telling us that you:

- Understand what you have read;
- Consent to take part in the research project;
- Consent to be involved in the research described;
- Consent to the use of your personal information as described.

What is this project about?

Tic disorders (TD) are neurodevelopmental disorders characterized by recurrent motor and/or vocal tics.TD are more common in males than in females. TD usually begin at the age of 6 on average and the maximum severity of tics tends to occur between 10 and 12 years of age. More than 50% individuals with TD have at least one comorbid/coexisting neurological problem that can further impact social and academic activities or employment. Early diagnosis and intervention are indispensable to affected children.

Qihuang needle is a newly acupuncture instrument developed from the Jiuzhen (Nine Needles) created in the ancient time, which has been widely praised in recent years with the advantages of applying less acupoints, good effect and simple manipulation. It has been applied in the management of TD and demonstrated good effectiveness and compliance. However, the mechanism underlying Qihuang needle therapy for TD remains unclear. Therefore, this study aims to investigate how Qihuang needle influences TD via multi-omics analysis.

Who are the people should not participate in this project?

Children with secondary tic disorders, or severe diseases of important organ systems, or other neurological problems, or other conditions that that may influence the gut microbiota.

What does my participation involve?

Your participation in this research project will involve a survey which will require you to sign a consent form, completing a set of questionnaires including demographics questions, risk factors associated with TD, information on therapies for TD and scales to measure tic severity and gastrointestinal symptoms. The survey should take you no longer than 20 minutes to complete.

Following the completion of the survey, you need to collect feces and blood samples then you will be allocated to one of the two groups:

1. If you are individuals with TD: receive eight Qihuang needle therapy sessions (no retention after needling manipulation) twice a week for four weeks from a licensed acupuncturist at Panyu Hospital of Chinese Medicine (Guangzhou, China) or Foshan Fosun Chancheng Hospital (Foshan, China)

You will be asked to report any adverse events happen during the whole treatment period.

You will receive scales assessment ,and need to collect feces and blood samples after completing 4-week treatment and a follow-up visit 12 weeks after completing 4-week treatment.

Or

2. If you are healthy individual: receive NO treatment over the 4-week trial time

Do I have to take part in this research project?

Your participation in this research project is voluntary. If you do decide to participate, you will be given a consent form to sign, and you will be given a copy of this information letter to keep. You are free to withdraw from the project at any time. If you decide to withdraw from the project after the data has been analyzed, your individual data will not be removed as it cannot be identified.

Your privacy

By signing the consent form, you agree with the research team to collect and use personal information for research project. Any information or samples obtained in connection with this research project that can identify you will remain confidential. When all survey responses are returned to the research team, all data are automatically de-identified, and you will not be identifiable by any of your responses to the survey.

Your information and samples will only be used for the purpose of this research project and it will only be disclosed with your permission, except as required by law.

It is anticipated that the results of this research project will be published and/or presented in a variety of professional forums. In any publication and/or presentation, the information will be provided in such a way that you cannot be identified, except where requested for specific reasons, and then you will be asked to provide written consent.

You have the right to request access to the information about you that is collected and stored by the research team. You also have the right to request that any information that you disagree to be corrected. Please inform the research team member named at the end of this letter if you would like to access your information.

All data collected will be kept in accordance with Good Clinical Practice Data Management Policy. Clinical data will be recorded in case report forms (CRFs) and will be imported into an electronic database. Electronic data will be stored on a secure Epi data. The original CRFs and other documents will be preserved for a period of five years and destroyed, if appropriate at the end of the retention period at South China Research Centre for Acupuncture and Moxibustion in Guangzhou University of Chinese Medicine. Samples will be retained for one year and destroyed.

Possible Benefits

We anticipate, based on past research, that acupuncture treatment that we will provide may be helpful in reducing severity of tic, increasing gut microbiome and quality of life. We hope that the results of our research can be used to inform our knowledge about how to better manage tic disorder that many children experience.

Possible reward

You will not get any reward for participating the research project. You will be free of registration, acupuncture treatment and scale assessment fee as compensation.

Possible Risks and Risk Management Plan

During the needling manipulation, you will feel soreness, numbness, distension, heaviness and so on around the point, which is normal needling sensation.

The common acupuncture-related adverse events are acupuncture syncope, sticking of the needle, bending of the needle, breaking of the needle, bleeding and hematoma. Slight bleeding and hematoma will be solved by local pressure. Acupuncture syncope will happen if the patient is weak and possesses nervous tension. If any accidents happen, needling will be stopped immediately and all the needles will be withdrawn. Patients will be asked to rest. Any adverse events will be monitored and recorded during

whole period of trial. If any adverse events happen between visits, you can consult the doctors.

There are no known risks to participating in this research project. If your health is impaired due to the research project, please inform your doctors and they will take appropriate medical measures. South China Research Centre for Acupuncture and Moxibustion in Guangzhou University of Chinese Medicine will pay the treatment fee and financial compensation according to relevant Acts.

What happens when this research study stops?

We will advise you of the outcomes via email communication. We also intend to publish our results in research journals and present them at research conferences locally, nationally and internationally. Your name or any other identifying information will not be included in any of the publications or presentations.

Has this research been approved?

This research project has received the approval of ethics committee of Panyu Hospital of Chinese Medicine（Ethic Approval Number:2022028）and Foshan Fosun Chancheng Hospital (Ethic Approval Number: CYIRB-LCYJ-2022006-PJ-20221227).

Contacts

If you would like to discuss any aspect of this project, please contact the following people.

**Chief Investigator**	Dr Chunzhi Tang
Adress	South China Research Centre for Acupuncture and Moxibustion, Guangzhou University of Chinese Medicine
Email	jordan664@163.com

**Coordinating investigator**	Yuyuan Tang
Adress	South China Research Centre for Acupuncture and Moxibustion, Guangzhou University of Chinese Medicine
Email	tangyuyuan@live.com

Participant Consent Form

Project title: Study on the mechanism of Qihuang Needle Therapy in children with tic disorders based on multi-omics
Approval Number: 2022028/ CYIRB-LCYJ-2022006-PJ-20221227
Trial registration No. ChiCTR2200057723
Principal Investigator: Dr Chunzhi Tang

I,___________________________________have read the Participant Information Letter. By signing this consent form, I acknowledge that I:

- have been provided with a copy of the Participant Information Letter, explaining the research study
- have read and understood the information provided
- have been given the opportunity to ask questions and have had questions answered to my satisfaction
- can contact the research team if I have any additional questions
- understand that participation in the research project will involve:
  - completion of a survey, collection of feces and blood samples
  - assigned into either a healthy control or treatment group receiving eight sessions of Qihuang needle therapy in four weeks from a licensed acupuncturist at Panyu Hospital of Chinese Medicine or Foshan Fosun Chancheng Hospital, and
  - completion of a survey which is the post-intervention questionnaire, collection of feces and blood samples
  - receiving a follow-up visit 12 weeks after completing four-week treatment.
- understand that the information provided will be kept confidential and that my identity will not be disclosed without consent
- understand that I am free to withdraw from further participation at any time, without explanation or penalty
- freely agree to participate in the project.
- The data and samples collected will be used only for this research project.

Participant name: ____

Signature: ______________________________ Date __________________
