# Supplementary material for: Mechanism of Qihuang needle therapy in the management of tic disorders: a clinical trial protocol
Source: Front Neurol. 2023 Apr 20;14:1036453. doi: 10.3389/fneur.2023.1036453 (PMC10157291; doi:10.3389/fneur.2023.1036453)
Supplement: Supplementary Material 2 — Machine learning method. [file Data_Sheet_2.pdf]

Supplementary File 2 Machine Learning Method

Data source and data preprocessing.

The 16S rRNA and serum metabolomics data used in this study were downloaded from the GEO or published work.

These data will be set as a source domain for deep neural network modeling. As for the target domain, we collect YGTSS score, GSI score and recurrence rate from case report forms of recruited participants. Multi-omics data is also included.

For the data, we select the expression features based on the features in the target domain data. We further removed samples with more than 20% missing values. We also filtered out samples missing clinical endpoint data. The data matrix was standardized such that each feature has a zero mean and unit standard deviation. To verify whether there are significant differences between the two groups of data, the ANOVA F value for each Multi-omics data was calculated for the training samples to select Multi-omics data as the input features for machine learning. The feature mask, ANOVA F value, and p values were calculated using the SelectKBest function (with the f_classif score function and k = the amount of omics data) of the python sklearn package(1).

Deep neural network modeling

We used the Theano python packages (http://deeplearning.net/ software/theano/) and Lasagne (https://lasagne. readthedocs.io/en/latest/) to train the DNN.

We used a pyramid architecture(2) with 6 layers: the number of nodes in the input layer is the feature number of omics data, 4 hidden layers including a fully connected layer with 256 nodes followed by a dropout layer, a fully connected layer with 128 nodes followed by a dropout layer, and a logistic regression output layer.

To fit a DNN model, we used the stochastic gradient descent method with a learning rate of 0.001 (lr = 0.001) to find the weights that minimized a loss function consisting of a cross-entropy and L2 regularization term:

$$\mathrm{l(W)} = -\sum_{i=1}^{n}(y_i \log(\hat{y}_i) + (1-y_i)\log(1-\hat{y}_i)) + \lambda_2||W||_2$$

where y_i is the observed label of patient i, $\hat{y}_i$ is the predicted label for patient i, and W represents the weights in the DNN.

Traditional activation functions such as the sigmoid and hyperbolic tangent functions have a gradient vanish problem in training a deep-learning model, which may lead to gradient decreasing quickly and training error propagating to forward layers. Here, we use the ReLU function f(x) = max (0, x), which is widely used in deep learning to avoid the gradient vanish problem. For each dropout layer, we set the dropout probability p = 0.5 to randomly omit half of the weights during the training to reduce the collinearity between feature detectors. To speed up the computation, we split the data into multiple mini-batches during training. We used a batch size of 20 (batch_size = 20) for the source domain as there was relatively large numbers of cases available for training. We set the maximum number of iterations at 100 (max_iter = 100) and applied the Nesterov momemtum(3) method (with momentum = 0.9 for each DNN model) to avoid premature stopping. The two regularization terms λ2 were

set at 0.001.

Transfer learning

For transfer learning, we set the 16S rRNA and serum metabolomics data downloaded from published data as the source domain and our study data as the target domain.

We first pretrain a DNN model using source domain data, without loss of generality, the pretraining process is to minimize the loss L over the source-domain data, resulting in the model parameter θ:

$$\underset{\theta}{\text{argmin}} = \sum_{(x,y)\in D_S} L_S(x, P(y|x), P_\theta(y|x))$$

which has the same architecture as described in the previous section. The training parameters are set as lr = 0.01, batch_ size = 20, p = 0.5, max_iter = 100, and momentum = 0.9. The pretrained model is considered as a starting point in the target domain. After the initial training, the DNN model will then be fine-tuned using backpropagation on the target domain data:

$$\underset{\theta'}{\text{argmin}} = \sum_{(x,y)\in D_t} L_T(x, P(y|x), P_\theta(y|x))$$

where θ' is the final model's parameter. In the fine tuning, the learning rate was set at 0.002 and the batch size was set at 5 as the model had been partially fitted and the target dataset was small.

The performance of the model

To see how well models is performing, we conduct 10-fold cross-validation on the source domain and at each iteration, 80% data will be used for training, 10% for validation, and 10% for testing. The results of the cross-validation folds were finally pooled to calculate the overall metrics, including accuracy, F1-score.

References

1. Pedregosa F, Varoquaux G, Gramfort A, Michel V, Thirion B, Grisel O, et al. Scikit-Learn: Machine Learning in Python. *the Journal of machine Learning research* (2011) 12:2825-30.

2. Phung SL, Bouzerdoum A. A Pyramidal Neural Network for Visual Pattern Recognition. *IEEE Trans Neural Netw* (2007) 18(2):329-43. doi: 10.1109/TNN.2006.884677.

3. Sutskever I, Martens J, Dahl G, Hinton G. On the Importance of Initialization and Momentum in Deep Learning. In: Sanjoy D, David M, editors. *Proceedings of the 30th International Conference on Machine Learning*; Proceedings of Machine Learning Research: PMLR (2013). p. 1139--47.
